# Corneal Confocal and Specular Microscopic Characteristics in Primary Open-Angle Glaucoma: An Optical Coherence Tomography (OCT)-Based Case-Control Study

**DOI:** 10.7759/cureus.76411

**Published:** 2024-12-26

**Authors:** Ali H Naqvi, Saad A Khan, Mahmood Ali, Muhammad A Moqeet, Hira Muazzam, Fahim Ullah Khan, Muhammad Saad, Waleed Ahmed, Farah Akhtar, Wajid A Khan

**Affiliations:** 1 Ophthalmology, Al-Shifa Trust Eye Hospital, Rawalpindi, PAK; 2 Cornea and Refractive Surgery, Al-Shifa Trust Eye Hospital, Rawalpindi, PAK; 3 Glaucoma, Al-Shifa Trust Eye Hospital, Rawalpindi, PAK

**Keywords:** biomechanics of the cornea, confocal microscopy, optical coherence tomography (oct), primary open-angle glaucoma, specular microscopy

## Abstract

Background: Glaucoma, particularly open-angle glaucoma (OAG), is a leading cause of irreversible blindness, associated with optic nerve damage, retinal ganglion cell death, and visual field defects. Corneal biomechanical properties and cellular components, such as corneal nerve and keratocyte densities assessed by in vivo confocal microscopy (IVCM), may serve as biomarkers for glaucoma progression. This study aimed to explore the relationship between corneal nerve parameters, keratocyte density, and optical coherence tomography (OCT)-derived retinal nerve fiber layer (RNFL) thickness in primary open-angle glaucoma (POAG) patients and controls.

Methods: This case-control study was conducted at Al-Shifa Trust Eye Hospital, Rawalpindi, Pakistan, from January 2023 to October 2024. It included 26 eyes of 17 glaucoma patients and 28 eyes of 18 age-matched controls. POAG was diagnosed based on elevated intraocular pressure (IOP), optic disc changes, RNFL defects, and visual field abnormalities. Participants underwent full ophthalmic evaluation, including OCT for RNFL thickness, specular microscopy, and corneal confocal microscopy (CCM). Data were analyzed using IBM SPSS Statistics for Windows, Version 26.0 (Released 2019; IBM Corp., Armonk, New York, United States), with Spearman's correlation and linear regression for association analysis.

Results: Anterior stromal keratocyte density was significantly lower in glaucoma patients (436.63±145.44 cells/mm²) compared to controls (546.54±141.20 cells/mm²; p=0.007). No significant difference was found in posterior stromal keratocyte density (p=0.788). Corneal nerve parameters showed a higher nerve fiber length in glaucoma patients (19.25±5.74 mm/mm²), but the difference was not significant (p=0.143). Nerve branch density was significantly higher in glaucoma patients (40.22±23.44 branches/mm²) compared to controls (26.12±10.17 branches/mm²; p=0.054). Specular microscopy revealed significantly lower endothelial cell density in glaucoma patients (2159.8±393.39 cells/mm²) compared to controls (2474.15±272.59 cells/mm²; p=0.002). OCT measurements showed a significantly thinner global RNFL in glaucoma patients (69.79±24.79 µm) compared to controls (98.86±9.04 µm; p<0.001).

Spearman's correlation analysis showed that anterior keratocyte density was positively correlated with global (r=0.294; p=0.045), superior (r=0.312; p=0.031), and inferior quadrant RNFL thickness (r=0.285; p=0.049). It was also negatively correlated with central corneal thickness (CCT) (r=-0.367; p=0.039). In multivariate analysis, the duration of glaucoma was significantly associated with RNFL thickness (p=0.011).

Conclusion: This study found that anterior stromal keratocyte density was positively correlated with RNFL thickness, suggesting a potential link between corneal cellular changes and glaucoma severity. Endothelial cell density was significantly lower in glaucoma patients, which may reflect the disease's impact or the effect of medications on corneal health. While corneal nerve parameters did not show significant differences, these findings highlight the importance of corneal biomechanical properties in glaucoma pathophysiology. Further studies with larger sample sizes and longer follow-up periods are needed to confirm these findings and explore the role of corneal parameters in glaucoma progression.

## Introduction

Glaucoma is a term for a group of diseases with progressive degeneration of the optic nerve and eventual death of retinal ganglion cells. Open-angle glaucoma (OAG) is a sub-type, commonly described as a chronic, progressive optic neuropathy characterized by an open angle, with optic nerve head (ONH) changes and visual field defects, usually associated with raised intraocular pressure (IOP) [1]. About 57.5 million people are affected by glaucoma worldwide, and it remains the second most common cause of irreversible blindness [2]. It is interesting that glaucoma can be found in people with a statistically normal range of IOP, termed normal-tension glaucoma (NTG) [3], hinting at some other significant pathology besides the raised pressure commonly associated with the disease. Some authors also consider it to lie on the same spectrum as primary open-angle glaucoma (POAG), which is characterized by raised IOP.

The Ocular Hypertension Treatment Study (OHTS) [4] was an important landmark that outlined a few risk factors for the progression of ocular hypertension to POAG, including age, cup-to-disc ratio, pattern standard deviation, IOP, and, importantly, central corneal thickness (CCT). Currently, genome-wide association studies have identified several genes/loci including ABCA1, MYOC, OPTN, and TBK1 for POAG and NTG to name a few [5]. Other factors, based on medical history, are important, as lower ocular perfusion pressure and anti-hypertensive medications along with systemic diseases have been associated with glaucoma [6]. Such diverse association outlines the multifactorial causation of the disease.

At the tissue level, there is substantial evidence in the literature highlighting the lamina cribrosa, a sieve-like structure occupying the posterior scleral canal through which ganglion cell axons pass, as a primary site of damage. This damage can be due to the stress and strain induced by the IOP and its fluctuations, more so with increasing age [7]. Moreover, it has been suggested that the vascular etiology associated with glaucoma may be related to the response of the lamina cribrosa deformity, related to its structure and material properties, in turn, affected by the composition of its extracellular matrix (ECM). This matrix undergoes remodeling along with the sclera, resulting in the varying response of these tissues to IOP changes [8].

Of particular interest is how the corneal biomechanical properties are associated with glaucoma regardless of CCT [9]. This biomechanical property can be assessed with the ocular response analyzer. This relatively newer technology can determine the corneal hysteresis (CH) as well as the corneal resistance factor (CRF), thus helping in IOP adjustment according to biomechanical properties [10]. Generally, these values are reduced in association with glaucoma, in both POAG and NTG. Moreover, CRF and CH are negatively correlated with keratocyte density [11]. Since lower CH and CRF have an important association with progressive optical coherence tomography (OCT) retinal nerve fiber layer (RNFL) thickness loss in glaucoma [12], it stands to reason that keratocyte density may play a significant role and, hence, warrant studies assessing its relationship with OCT RNFL thickness. This is a relatively unexplored territory to the best of our knowledge.

One way of examination of the corneal tissue is by using in vivo confocal microscopy (IVCM). This allows the quantification of the sub-basal nerve plexus and allows the visualization of the live keratocytes throughout the stroma, in real-time. Some studies have shown a reduced keratocyte density in glaucoma [13], but these usually focus on the effects of topical medications. Furthermore, in an IVCM study on the participants of the OHTS, the effects of chronic anti-glaucoma medications were evident on sub-basal nerves only, with keratocytes and corneal endothelial cell characteristics not showing any significant change. However, these patients were ocular hypertensives, without any suspicious optic discs [14]. A similar effect on the corneal nerve density by topical medications has been seen in other studies as well [15]. More recent studies involving IVCM reveal an increase in corneal nerve fiber length (NFL) along with other characteristics in NTG compared to POAG [16], and these parameters have been correlated with RNFL changes in both types [17].

The goal of this study is to elucidate associations between corneal nerves, corneal endothelial characteristics, and, importantly, stromal keratocyte density, with OCT RNFL parameters. One potential future treatment modality currently in the experimental stages is the use of lasers in collagen remodeling near the lamina cribrosa [18]. Thus, if OCT parameters are found to be associated with keratocyte cell density (CD), future studies could investigate the possible role of cellular as well as extracellular components of the corneal tissue in risk stratification.

## Materials and methods

The current case-control study was carried out at the Al-Shifa Trust Eye Hospital (ASTEH), Rawalpindi, Pakistan, from January 5, 2023, to August 10, 2024, using consecutive sampling after obtaining ethical approval from the institute's Ethical Review Committee on January 2, 2023 (approval number: ERC-02/AST-23). The study was deemed in line with the principles of the Declaration of Helsinki. Informed consent was taken from the patients before data collection.

Inclusion and exclusion criteria

All patients referred to the Glaucoma department were included in the study, after being assessed by a glaucoma consultant. POAG was defined as pre-treatment IOP of >21 mmHg, with open angles on gonioscopy, characteristic optic disc changes, RNFL defects, and/or progression on OCT or the presence of characteristic visual field defects, in the absence of secondary causes. Aged-matched controls were taken as normal healthy individuals scheduled for routine cataract surgery without any other ophthalmic diseases or prior history of surgeries.

Individuals with prior ocular trauma, contact lens use, ocular surgeries, spherical equivalent refractive error larger than ±5 diopters [19], corneal opacities and degenerations, diabetes, presence of any retinopathy, demyelinating diseases, and neurological defects were excluded.

Data collection and image analysis

All the participants underwent a full ophthalmic evaluation by a consultant ophthalmologist, followed by OCT RNFL testing for both eyes using the SPECTRALIS OCT (Heidelberg Engineering, Inc., Heidelberg, Germany), by a single experienced user. Specular microscopy (EM-4000, Tomey Corporation, Nagoya, Japan) was carried out, followed by corneal confocal microscopy (CCM) using the Rostock Cornea Module on a Heidelberg Retinal Tomograph (HRT) 3 (Heidelberg Engineering, Inc., Heidelberg, Germany).

Imaging of the corneal tissue was performed using the Cornea Module of the HRT 3 machine (Heidelberg Engineering, Inc., Heidelberg, Germany). Proparacaine hydrochloride (0.5%) drops were used on both eyes, along with a coupling agent which was also used on the objective lens before applying the applanation cap and ensuring the absence of air bubbles. Patients were instructed to focus on the fixation light, and the onboard camera was used to monitor the eye's fixation. In the section mode of CCM, images were taken using a 400 µm field lens. The images were taken from the sub-basal nerve plexus of the central cornea, the anterior stroma, defined as the anterior 33% of stromal depth, and the posterior stroma, defined as the posterior 33% of the stromal thickness [20]. A total of three images were selected for each region for a total of nine images for each eye. All data collection was carried out by a single examiner. Nerve parameters were assessed using a validated nerve tracing package, CCMetrics, which has been used in various studies [21]. Keratocyte density was assessed by counting the cells on the open-source software, ImageJ [22], and taking mean values for the images.

Outcome variables

The primary objective of the study was to assess keratocyte density in both cases and controls and their association with the OCT RNFL thickness. The keratocyte density was calculated separately for the anterior and posterior stroma. In addition, nerve parameters such as NFL in mm/mm^2^, nerve fiber density (NFD) in fibers/mm^2^, and nerve branch density (NBD) in branches/mm^2^ were also assessed. Specular microscopy variables included endothelial CD, CCT, coefficient of variation (CV) of cell size, standard deviation (SD) of mean cell area, and percentage of hexagonal cells (HEX %, 6A).

Data analysis

Data were entered on IBM SPSS Statistics for Windows, Version 26.0 (Released 2019; IBM Corp., Armonk, New York, United States). Quantitative variables were expressed as mean±SD where applicable, and qualitative data were expressed as frequencies and percentages. The normality of the continuous data was assessed before applying statistical tests, including the independent t-test and Pearson's correlation for normally distributed data and Spearman's correlation for data not found to be normally distributed. Finally, linear regression analysis was used, and for all tests, a p-value of <0.05 was taken to be statistically significant.

## Results

Patient characteristics

In the final analysis, 26 (48.1%) eyes of 17 glaucoma patients (14 males: 82.35% and three females: 17.64%) and 28 (51.9%) eyes of 18 controls (14 males: 77.77% and four females: 22.22%) were included. The mean ages of cases and controls were 64.29±12.24 and 65.28±8.37 years, respectively, which were not statistically different as assessed by the t-test (p=0.782). The median duration of glaucoma evaluated using the hospital records for these patients was five months (range: 0-72 months) as shown in Table 1. The mean number of anti-glaucoma topical agents being used by the glaucoma group was 2.61±1.46, with 12 of the 18 individuals using prostaglandin analogues (Table 1).

**Table 1:** Demographic characteristics 1: Fisher's exact test; 2: independent t-test (t=-0.279); 3: median (range); *: mean±SD

Characteristics		Cases	Controls	P-value
Gender (n(%))	Males	14 (50%)	14 (50%)	1.00^1^
	Females	3 (42.8%)	4 (57.2%)	
	Total	17 (48.6%)	18 (51.4%)	
Age (years)*		64.29±12.24	65.28±8.37	0.782^2^
Duration of glaucoma (months)		5^3^ (0-72)	-	-

Confocal microscopy parameters between cases and controls

The keratocyte density for the anterior stroma was 436.63±145.44 cells/mm^2^ among cases vs. a mean of 546.54±141.20 cells/mm^2^ in controls, with the independent t-test being statistically significant (p=0.007). The keratocyte density in the posterior stroma was not statistically different (p=0.78).

The mean NFL for cases was 19.25±5.74 mm/mm^2^ and 17.00±3.90 mm/mm^2^ for the controls. The NFD was 29.89±10.14 fibers/mm^2^ and 30.11±7.74 fibers/mm^2^, and the NBD was 40.22±23.44 branches/mm^2^ and 26.12±10.17 branches/mm^2^, for cases and controls, respectively (Mann-Whitney U test; p=0.054). Corneal nerve parameters and keratocyte densities are summarized in Table 2.

**Table 2:** Confocal microscopy characteristics of cases and controls 1: independent samples t-test; 2: Mann-Whitney U test; *: statistically significant; a: data is presented as mean±SD

Confocal microscopy parameters^a^	Cases	Controls	P-value (test statistics)
Nerve fiber length	19.25±5.74	17.00±3.90	0.143^1^ (1.495)
Nerve fiber density	29.89±10.14	30.11±7.74	0.937^1^ (-0.079)
Nerve branch density	40.22±23.44	26.12±10.17	0.054^2^ (160)
Anterior stroma keratocyte density	436.63±145.44	546.54±141.19	0.007^1*^ (-2.82)
Posterior stroma keratocyte density	233.67±47.22	237.13±45.33	0.788^1^ (-0.27)

Specular microscopy parameters between cases and controls

The CCT did not differ statistically between cases and controls (513.57±33.94 vs. 492.94±35.1; p=0.105). The endothelial CD showed a significant difference between the groups, with cases having a mean of 2159.8±393.39 cells/mm^2^, while for the controls, it was 2474.15±272.59 cells/mm^2^ (Mann-Whitney U test; mean rank 11.80 vs. 22; p=0.002). Further parameters for specular microscopy are summarized in Table 3.

**Table 3:** Specular microscopy characteristics of cases and controls 1: independent samples t-test; 2: Mann-Whitney U test; *: statistically significant; a: data is presented as mean±SD

Specular microscopy parameters^a^	Cases	Controls	P-value (test statistics)
Central corneal thickness	513.57±33.94	492.944±35.10	0.105^1^ (t=1.673)
Cell density	2159.80±393.3	2474.15±272.58	0.003^2*^ (U=228)
Standard deviation of mean cell area	201.6±103.8	156.05±32.31	0.136^2^ (U=88)
Coefficient of variation of cell size	39.80±8.44	38.11±4.48	0.805^2^ (U=121)
Percentage of hexagonal cells (6A)	46.4±7.44	48.35±6.34	0.429^1^ (t=-0.801)

OCT RNFL values

The global OCT RNFL values along with superior, inferior, nasal, and temporal quadrants for the cases were 69.79±24.79 µm, 82.96±36.30 µm, 82.16±34.36 µm, 59.7±30.62 µm, and 48.9±14.55 µm, respectively, while for the controls, these were 98.86±9.04 µm, 118.5217±10.82 µm, 126.26±13.71 µm, 78.96±9.31 µm, and 71.74±26.11 µm, respectively.

Relationship of IVCM with RNFL in glaucoma

Spearman's correlation was used to assess the association of RNFL thickness values with corneal parameters. Anterior keratocyte density was positively correlated with global (Spearman's r=0.294; p=0.045), superior quadrant (Spearman's r=0.312; p=0.031), and inferior quadrant (Spearman's r=0.285; p=0.049) RNFL thickness values. The anterior keratocyte density was negatively correlated with the CCT value (Pearson's r=-0.367; p=0.039).

Sixteen (61.5%) of the 26 eyes were on topical prostaglandin analogues, and there was a statistically significant increase in CCT among those using the drug (525.7±32.39 vs. 483.25±11.35 µm). Endothelial cell counts were observed to be higher in those using these analogues (2332.6±129.26 cells/mm^2^ vs. 1684.5±505.71 cells/mm^2^); however, the sample for this analysis was small (n=4, not using prostaglandin).

Finally, a regression model was used to predict the global RNFL value, including those variables that showed a trend for significance in the univariate model. Only the duration of glaucoma was found to be significant in the multivariate analysis (Table 4).

**Table 4:** Linear regression for the outcome variable of global retinal nerve fiber layer value *: statistically significant

Univariate analysis			Multivariate analysis		
Variable	Standardized beta	P-value	Standardized beta	P-value	95% CI
Anterior keratocyte density	0.277	0.059	-0.442	0.098	-0.163 to 0.016
Duration of glaucoma	-0.635	0.001	-0.846	0.011	-1.428 to -0.237
Cell density on specular microscopy	0.321	0.074	0.007	0.987	-0.059 to 0.060
Coefficient of variation on specular microscopy	-0.319	0.086	-0.316	0.387	-3.208 to 1.357

## Discussion

IVCM is used in various industries including medicine and biology. Importantly, it is a useful tool for the visualization of live corneal tissue, including sub-basal nerve plexus, keratocytes, and endothelial cells, in the clinical setting. It can help visualize pathogens in some forms of keratitis [23] and has a role in corneal disease as an adjunctive investigation, providing en face, real-time imaging. Currently, three types of confocal imaging platforms for the cornea are available, with the Heidelberg Retinal Tomograph with the Rostock Cornea Module (HRT-RCM) being superior in resolution to previous models and more widely used by clinicians and researchers [24]. Patient examination takes around 10-15 minutes, but the imaging is unique and provides important information to the clinician. Other methods of keratocyte visualization include the use of a fluorescein camera and even the use of automated ultra-high-resolution OCT to quantify keratocyte density [25]. Some custom machines allow faster and more accurate volumetric analysis, but the technology is still relatively new. Hence, currently available and accessible technology for in vivo corneal tissue assessment remains the IVCM.

The present case-control study included 26 eyes of 17 cases and 28 eyes of 18 age-matched controls. Corneal confocal images were acquired to assess sub-basal corneal NFL, NFD, NBD, and keratocyte densities in the anterior and posterior stroma. Specular microscopy was done to assess quantitative and qualitative endothelial characteristics, and an OCT scan of the RNFL thickness around the optic disc was obtained in all participants. The keratocyte density in the anterior stroma (up to 100 µm below the basal epithelium) was significantly lower among cases compared to controls. Further, the endothelial CD was significantly lower in the glaucoma group, but other specular characteristics were similar to the control group. Applying correlation tests, the anterior keratocyte density was positively associated with the global, superior, and inferior RNFL values, while it was inversely related to the CCT. The eyes receiving prostaglandin analogues had a statistically elevated CCT value, compared to other glaucoma patients not using these agents. In the univariate analysis, keratocyte density, endothelial CD, and endothelial CV value showed a trend towards significance, but only the duration of glaucoma was significant, which remained the only significant factor in the multivariate model. The lack of an association of keratocyte density in the multivariate model may be due to the small sample but still shows a trend, which needs to be elucidated by other, higher-powered studies. 

As mentioned previously, some studies have established that the sub-basal nerve plexus is reduced in glaucoma patients and that too may be due to the therapy itself [15]. This may be the reason why, in our study, we failed to demonstrate any nerve parameter changes between cases and controls, as both old and new glaucoma patients were enrolled, with a median time of five months only. This contrasts with some other studies, which use samples of patients with established diseases, and who are on treatment for several years, before inclusion in the study. A lower endothelial CD is associated with glaucoma [26] which is in line with the present study. This effect has been attributed to the toxic effects of glaucoma medications or to the high IOP. However, the other specular parameters mentioned previously were not significantly different. This may be in line with the previous study showing that endothelial characteristics along with other factors might not be affected [14].

The use of prostaglandin analogues has garnered particular attention as it seems to modify the stromal structure, with some studies suggesting a decrease in CCT, while others report an increase [27]. Their effect remains to be validated, but the cited meta-analysis shows that the effect is probably to decrease CCT, possibly by activating matrix metalloproteinases (MMPs). In our study, the CCT value was found to be increased in those using these agents (525.7±32.39 vs. 483.25±11.35; p=0.028), which is in line with the notion that prostaglandin might indeed exert effects on the stromal matrix. Moreover, prostaglandins have been shown to increase keratocyte densities [20] by similar mechanisms, and although our study did not show any significant results in this regard, it must be remembered that previous studies in the literature have used larger sample sizes with longer use of these agents. Hence, our study might not be powerful enough to elucidate these findings. Furthermore, some studies report a decrease in keratocyte density [13]. However, the latter study did not establish a glaucoma group that was not using these agents as a comparison, to establish the effect on keratocytes being truly due to this class of drugs. If such an association exists, the impact on keratocyte density and CCT by prostaglandins will need to be controlled by future studies to make clear associations with other parameters and glaucoma. It is worth mentioning here that considering the conflicting reports of their effect, the effect size of prostaglandins may probably be small.

In the present study, finding a positive correlation between anterior keratocyte density and global RNFL values on OCT, in the absence of a correlation with CCT, seems interesting. Even though landmark studies have demonstrated CCT as an independent risk factor for glaucoma progression, in our sample, the lower anterior keratocyte density among cases was showing a trend towards significance, which disappeared on the multivariate model. However, CH and CRF, as previously mentioned [10], have been found to possibly be more sensitive to glaucoma progression compared to CCT, and lower values confer an increased risk. However, lower CH and CRF values have been negatively associated with keratocyte density in healthy eyes [11], meaning that glaucoma progression would be associated with an increased keratocyte density. This seems to contrast with our study but highlights the importance of the biomechanical properties of the corneal stroma, being due to not just the stromal matrix but cellular components as well.

There is evidence that the deformation of the lamina cribrosa at the posterior scleral canal is due to the individual variation of its tissue components [8], conferring relative susceptibility to the IOP among other factors. Keeping in view the association of CH and CRF with glaucoma, along with the role of CCT, the finding of keratocyte changes may be relevant as one of the markers or predictors of the susceptibility of the lamina cribrosa to IOP and hence loss of RNFL by mechanical deformation of the nerve fibers passing through the sieve-like structure. The role of keratocyte density and perhaps morphology may be important study parameters for future studies, as such data is scarce in the literature to the best of our knowledge. Perhaps keratocyte density is indeed decreased in glaucoma, but this notion warrants more studies with larger sample sizes, for testing hypotheses, such as lower keratocyte density being a proxy marker for lower tissue rigidity of the lamina cribrosa and, hence, increased deformability of the structure. To assess this hypothesis, studies on at-risk individuals, such as those having a family history of glaucoma or those with confirmed genetic susceptibility, should be done, comparing those without the risk. Even though the RNFL would not be affected without glaucoma, the corneal cellular and extracellular components may have precursor changes. Finally, there is evidence that activated keratocytes may release growth factors for corneal nerve regeneration [28]. This may have a role in glaucoma which warrants further studies; however, the current view is that the decrease in nerve density seems to be the effect of anti-glaucoma medications [15].

As mentioned previously, a potential treatment may lie in strengthening the extracellular components of the lamina cribrosa [18]. This is currently in the experimental stages using lasers and not in routine practice. Nevertheless, it outlines the need for reassessing corneal parameters for risk assessment and possible prognosis if such an intervention becomes feasible.

The strength of the study includes the use of a relatively new modality of IVCM, in POAG patients, as such data is lacking in this part of the world to the best of our knowledge and the technology is not widespread. Secondly, efforts were made to ensure age matching of cases and controls. Nevertheless, several limitations must be kept in mind when interpreting the results of this study. For one, a single observer analyzed the confocal images, and to assess agreeability, at least two observers should independently analyze the images to increase the reliability of the results. Secondly, as already mentioned, people on long-standing medications need to be recruited to assess the effects on corneal nerve parameters, as it seems early in the course, these might not be affected. Future studies with larger sample sizes, and recruitment of patients with longer duration of glaucoma and its treatment, and perhaps the use of OCT technology for the quantitative analysis of the optic nerve structure itself, will add to the still-growing body of knowledge regarding corneal parameters in POAG.

## Conclusions

Anterior stromal keratocyte density may be positively associated with RNFL thickness values on OCT, but conclusive evidence remains to be elucidated by future studies with larger sample sizes and long-term topical anti-glaucoma drug use, as it may influence corneal parameters. Perhaps earlier in the course of treatment, corneal nerve parameters are not affected, unlike endothelial cell parameters, as seen in the present study.
